# T‐Wave Alternans in Nonpathological Preterm Infants

**DOI:** 10.1111/anec.12745

**Published:** 2020-01-27

**Authors:** Ilaria Marcantoni, Agnese Sbrollini, Gloria Agostinelli, Francesca Chiara Surace, Massimo Colaneri, Micaela Morettini, Marco Pozzi, Laura Burattini

**Affiliations:** ^1^ Department of Information Engineering Università Politecnica delle Marche Ancona Italy; ^2^ Department of Paediatric and Congenital Cardiac Surgery and Cardiology Ospedali Riuniti Ancona Italy

**Keywords:** arrhythmias‐risk index, heart‐developing marker, heart‐rate adaptive match filter, preterm infant, sudden infant death syndrome, T‐wave alternans

## Abstract

**Background:**

Sudden infant death syndrome is more frequent in preterm infants (PTI) than term infants and may be due to cardiac repolarization instability, which may manifest as T‐wave alternans (TWA) on the electrocardiogram (ECG). Therefore, the aim of the present work was to analyze TWA in nonpathological PTI and to open an issue on its physiological interpretation.

**Methods:**

Clinical population consisted of ten nonpathological PTI (gestational age ranging from 29$3\;/\;7$ to 34$2\;/\;7$ weeks; birth weight ranging from 0.84 to 2.10 kg) from whom ECG recordings were obtained (“Preterm infant cardio‐respiratory signals database” by Physionet). TWA was identified through the heart‐rate adapting match filter method and characterized in terms of mean amplitude values (TWAA). TWA correlation with several other clinical and ECG features, among which gestational age–birth weight ratio, RR interval, heart‐rate variability, and QT interval, was also performed.

**Results:**

TWA was variable among infants (TWAA = 26 ± 11 µV). Significant correlations were found between TWAA versus birth weight (*ρ* = −0.72, *p* = .02), TWAA versus gestational age–birth weight ratio (*ρ* = 0.76, *p* = .02) and TWAA versus heart‐rate variability (*ρ* = −0.71, *p* = .02).

**Conclusions:**

Our preliminary retrospective study suggests that nonpathological PTI show TWA of few tens of µV, the interpretation of which is still an open issue but could indicate a condition of cardiac risk possibly related to the low development status of the infant. Further investigations are needed to solve this issue.

## 1 INTRODUCTION

Preterm birth, defined as a birth of an infant born alive before the 37^th^ week of pregnancy is completed ("World Health Organization, 2018") does not represent a so rare phenomenon ("World Health Organization, 2018"). According to the World Health Organization, every year about 15 million (from 5% to 18% across 184 countries) infants are preterm and this number is rising ("World Health Organization, 2018"). Moreover, preterm birth complications are among the first causes of demise among under 5‐year‐old children ("World Health Organization, 2018"). Despite of this, preterm infants (PTI) have not been so much studied until now and knowledge about developmental biology and disease phenomena is still limited (Engle et al., 2007). PTI are physiologically immature and compensatory responses to the extrauterine environment are reduced if compared with term infants; for this reason, their morbidity and mortality risks are higher (Engle et al., 2007).

Among other diseases, PTI have higher risk of developing sudden infant death syndrome (SIDS) than infants born at term (Rohana, Ishak, & Wan Nurulhuda, 2018; Thompson & Mitchell, 2006). SIDS was formalized about 50 years ago and is defined as the sudden death of an apparently healthy infant under 1 year that cannot be explained despite investigations, including autopsy, death scene examination, and knowledge of the infant's clinical history (Haas, 2018; Randall, Thompson, & Wilson, 2019; Rohana et al., 2018). SIDS is considered among the first causes of death during infancy (Kojima et al., 2018). Nevertheless, the exact mechanism underlying SIDS remains obscure and its etiology is likely multifactorial (Idriss, Van Hare, Fink, & Rosenbaum, 2002; Rohana et al., 2018; Schwartz et al., 1998). Apart from risk factors linked to infant living habits and parental practices (like way of sleeping or exposure to cigarette smoking), anamnestic data (like prematurity or low weight at birth), physiological abnormalities (like abnormalities of the central nervous system, abnormalities of the respiratory system, abnormalities of the cardiovascular system, or a combination of them) have been accounted as the main causes for the onset of this disease (Idriss et al., 2002; Rohana et al., 2018).

Studies on cardiac causes for SIDS have been focused on arrhythmia vulnerability attributable to repolarization instability (Idriss et al., 2002). At birth, cardiac electrophysiologic function is typically not complete (Idriss et al., 2002) and the dynamics of cardiac repolarization is altered with respect to adults (Idriss et al., 2002; Kojima et al., 2018; Pishva & Khosrojerdi, 2003). For example, it has been observed that QT variability may serve as an index of the maturity of the cardiac autonomic nervous system and myocardial depolarization (Kojima et al., 2018), while QT dispersion may be used as a prognostic factor for estimation of neonatal mortality (Pishva & Khosrojerdi, 2003). Few experimental studies on animals have suggested that repolarization alterations at birth may also manifest as T‐wave alternans (TWA) (Idriss & Bell, 2008; Idriss et al., 2002), a phenomenon consisting in the beat‐to‐beat fluctuation of JT morphology in the electrocardiogram (ECG) at stable heart rate. Often, TWA occurs at microvolt level, and thus, it is not visible at the naked eye so that computerized approaches are needed for its identification (Bini & Burattini, 2013; Burattini, Bini, & Burattini, 2009, 2010). TWA is widely studied in adult ECG due to its link with severe ventricular arrhythmias leading to sudden cardiac death (Rosenbaum, Albrecht, & Cohen, 1996). TWA in children is much less studied, and its interpretation is still far from being established (Idriss & Bell, 2008; Idriss et al., 2002; Makarov & Komoliatova, 2010; Makarov et al., 2010). For aught we know, only a couple of studies have analyzed TWA in human infant ECG (Makarov & Komoliatova, 2010; Makarov et al., 2010). In 2010, Makarov et al. (2010) analyzed TWA in twenty apparently healthy newborns in the first, second, and fourth day after birth and observed a peak level of TWA in the second day, possibly reflecting a restructuring of heart activity in the developing heart (in any case, TWA never exceeded 55 µV). According to Madias (2010), TWA changes in these newborns may be linked to changes in the T‐wave amplitude and do not necessarily reflect propensity to malignant ventricular arrhythmias (Madias, 2010). As far as we know, there is no study in literature about TWA on PTI. Therefore, the aim of the present work was to identify and quantify TWA in PTI and to evaluate association of TWA with infants’ clinical features at birth and with other ECG features that have been found or assumed to be associated to adult TWA (Burattini, Zareba, & Burattini, 2012; Hanna & Ahmed, 2013; Madias, 2007; Rashba et al., 2002; Zareba, Moss, Cessie, & Hall, 1994). The final purpose is to gain more insights into the TWA phenomenon in PTI and, in particular, to open an issue on the possibility of considering TWA in nonpathological PTI (and possibly in newborns in general) as a heart‐developing marker rather than an arrhythmias‐risk index.

## 2 METHODS

### 2.1 Preterm Infant Population

The study population was constituted by ten nonpathological PTI from the “Preterm infant cardio‐respiratory signals database” by Physionet (Gee, Barbieri, Paydarfar, & Indic, 2017; Goldberger et al., 2000). Data referred to the population were acquired and collected in the neonatal intensive care unit (NICU) at the University of Massachusetts Memorial Health care. PTI have a gestational age (GA) from 29$3\;/\;7$ to 34$2\;/\;7$ weeks (mean: 31$1\;/\;7\;$ ± 1$3\;/\;7$ weeks, Table 1) and birth weight (BW) from 0.84 to 2.10 kg (mean: 1.47 ± 0.41 kg; Table 1). Consequently, GA/BW ratio (days/kg), indicating the correct infant growth with respect to gestational age, ranged from 108 to 251 days/kg. The infants could spontaneously breath and did not present any congenital or perinatal infection of the central nervous system, intraventricular hemorrhage of grade II or higher, and hypoxic–ischemic encephalopathy. Hospitalization of the infants is justified by their condition of prematurity, which makes nursing daily monitoring necessary until their weight guarantees complete physical and physiological development, fundamental for an autonomous and risk‐free growth. A single channel of a three‐lead ECG signal was acquired (sampling frequency: 500 Hz; acquisition band‐pass cutoff frequencies: 0.5–55.0 Hz) when available from bedside patient monitors (Intellivue MP70, Philips Medical Systems) from 20.3 to 70.3 hr per infant. The choice of the available ECG was dependent on nursing preference. As far as the description of the database includes, during the acquisition there were no other interferences from equipment generating a signal close to a multiple of the T‐wave alternans frequency, like a breathing or feeding pump.

**Table 1:** Clinical features at birth of each preterm infant and corresponding mean ± standard deviation values over the entire population (Tot)

PTI	GA (weeks)	BW (kg)	GA/BW (days/kg)
1	29 $3\;/\;7$	1.20	172
2	30 $5\;/\;7$	1.76	122
3	30 $5\;/\;7$	1.71	126
4	30 $1\;/\;7$	0.84	251
5	32 $2\;/\;7$	1.67	135
6	30 $1\;/\;7$	1.14	185
7	30 $1\;/\;7$	1.11	190
8	32 $3\;/\;7$	2.10	108
9	30 $4\;/\;7$	1.23	174
10	34 $2\;/\;7$	1.90	126
Tot	31$1\;/\;7$ ± 1 $3\;/\;7$	1.47 ± 0.41	159 ± 44

Abbreviations: BW, birth weight; GA, gestational age; PTI, preterm infants.

Data acquisition was approved by the University of Massachusetts School Institutional Review Board of human subjects. In any case, all data from Physionet have been fully de‐identified and may be used without further independent ethics committee approval.

### 2.2 T‐wave alternans identification

**Figure 1:**
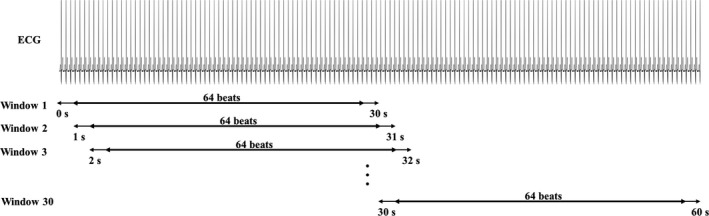
Electrocardiogram (ECG) windowing procedure

Identification and quantification of TWA on ECG recordings from PTI were performed through the heart‐rate adapting match filter (HRAMF) method (Burattini, Zareba, & Burattini, 2008). Approximately, only the first available minute of each ECG recording was analyzed. Specifically, overlapping 30‐s ECG tracings were recursively extracted every second for 30 times, overall covering 60 s of ECG. In each extracted tracing, the central 64‐beat ECG window was considered for the analysis of TWA (Figure 1). Then, each ECG window was prefiltered with a band‐pass bidirectional 6^th^‐order Butterworth filter (cutoff frequencies: 0.8 and 35.0 Hz) to remove low‐frequency interferences due to respiration and the high‐frequency interferences due to power line. Baseline was estimated as a 3^rd^‐order spline interpolation of fiducial points sited 50 ms before R peaks and then subtracted (Burattini, Bini, & Burattini, 2011). Then, each ECG window was tested to assess its suitability for TWA analysis. Specifically, an ECG window was considered suitable for TWA analysis if characterized by a stable heart rate and good quality signal. Heart rate was considered stable if the standard deviation of the RR intervals was at most 10% the RR‐interval median value (Burattini et al., 2011). Instead, signal quality was considered good if there were at most 5 actual beats in the ECG window that needed to be replaced since poorly correlating (correlation coefficient <0.70) with the median beat, which was computed over all the beats included in the considered window (Burattini et al., 2011). Only ECG windows which were found to be suitable for TWA identification were submitted to the HRAMF (Burattini et al., 2008).

**Figure 2:**
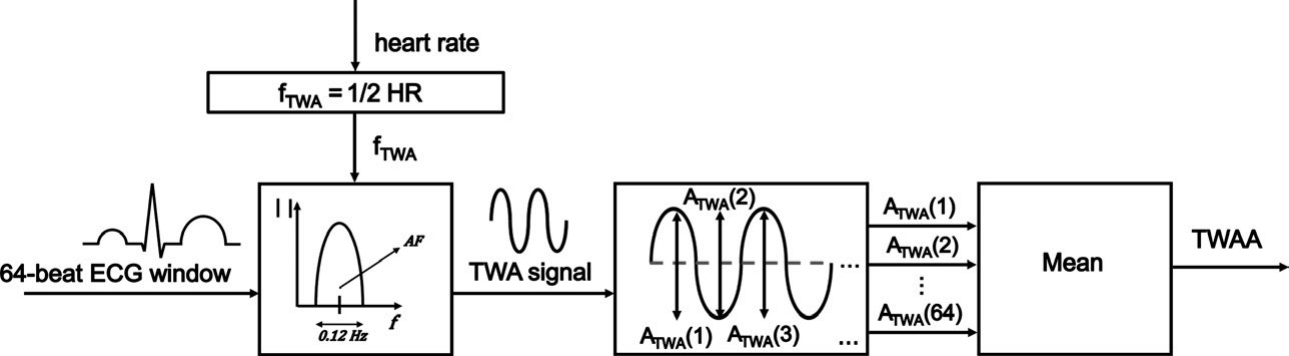
Procedure of the heart‐rate adaptive match (HRAMF) filter

The theoretical approach of the HRAMF method is based on the assumption that, in the speculative condition of a perfectly constant heart rate, TWA is a phenomenon characterized by a specific frequency, *f*_TWA_, by definition equal to half heart rate. However, some physiological heart‐rate variability (HRV) always occurs in any real ECG window so that the method actually considers TWA featured by a narrow frequency band, rather than a single frequency. Technically, the HRAMF implementation consists of a 6^th^‐order bidirectional Butterworth band‐pass filter characterized by a passing band that is 0.12 Hz wide (Burattini et al., 2008) and centered at the specific *f*_TWA_ of the analyzed ECG window (Figure 2). ECG windows characterized by different heart rate will consequently be characterized by different *f*_TWA_ values, to which the HRAMF adapts to set its passing band. The input signal of HRAMF is the preprocessed ECG window. When HRAMF is fed with it, it computes heart rate, corresponding *f*_TWA_ and passing band. Then, it filters out every ECG component not pertaining to the TWA frequency band.

In the presence of TWA, the HRAMF output signal is the TWA signal, a constant phase pseudo‐sinusoidal signal, possibly amplitude modulated, with its maxima and minima over the T wave; otherwise, the TWA signal is a zero constant (Burattini et al., 2008). To localize these maxima and minima, a windowing of the T wave was necessary, but for TWA detection aim, the exact identification of the T‐wave endpoints is not crucial. T‐wave windowing was performed using and adapted version of the experimental formulae previously proposed for adults (Burattini, Zareba, & Moss, 1999). Specifically, T‐wave onset is located 36 ms, 60 ms, and 90 ms after the R peak if RR < 0.36 s, 0.36–0.66 s, and > 0.66 s, respectively; T‐offset is located basing on T‐wave width, computed as $\text{0.32}\times \sqrt{\text{(}meanRR)}$. These formulas were obtained by considering that the heart rate of a premature infant (similarly to a fetus at the end of the gestational age) is, on average, half that of an adult (Marcantoni et al., 2018, 2017). A local estimate of TWA amplitude (A_TWA_; µV), associated with each single beat, is directly given by twice the TWA signal amplitude in correspondence of the T wave (Figure 2). In order to have TWA features globally representing all 64 beats in a window, mean value of the 64 A_TWA_ values was computed, besides amount of accepted ECG windows (NW). Eventually, median values of mean TWA amplitude over the NW ECG windows recognized as suitable for TWA analysis was computed and indicated as TWAA (µV). If NW < 24 (80% of total windows), TWA analysis was considered unreliable, and the TWA identification procedure was repeated for the next following minute in the ECG tracing.

### 2.3 Additional electrocardiographic characterization

The 1‐min ECG tracing used to perform TWA analysis was also characterized in terms of the following other common ECG features, which in adults were previously found or hypothesized to correlate with TWA (Burattini et al., 2012; Hanna & Ahmed, 2013; Madias, 2007; Rashba et al., 2002; Zareba et al., 1994) : median RR interval (RR, ms); median heart rate (HR, bpm); HRV (ms, measured as RR standard deviation); median T‐wave amplitude (TA, µV); median QRS‐complex duration (QRSD, ms); median QT‐interval duration (QT, ms); and median corrected QT‐interval duration (QTc, ms) computed according to Bazett's formula (Bazett, 1920).

### 2.4 Statistics

Distribution of TWAA, clinical features at birth (GA, BW, and GA/BW) and additional ECG features (RR, HR, HRV, TA, QRSD, QT, and QTc) over the population were described in terms of mean ± standard deviation. Possible associations between TWAA variable and both clinical features and additional ECG features were analyzed through the nonparametric Spearman rank (given the low number of our PTI) correlation coefficient (*ρ*). Statistical significance (*p*) was set at .05 in all cases.

## 3 RESULTS

**Figure 3:**
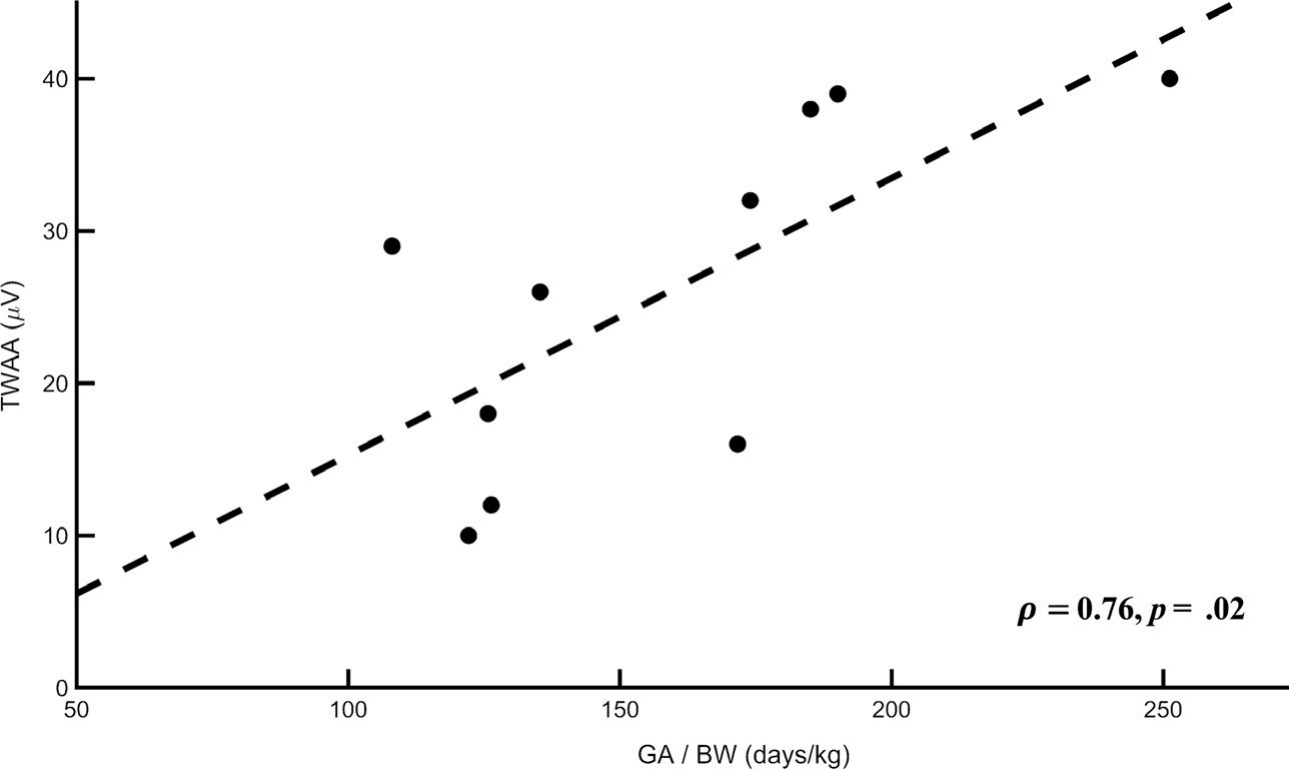
Regression line between mean T‐wave alternans amplitude (TWAA, µV) and gestational age–birth weight ratio (GA/BW, days/kg)

TWA features and additional ECG features relative to the analyzed population of PTI are reported in Table 2: 6 PTI out of 10 had NW = 30 (100% of windows accepted for TWA analysis), 2 PTI out of 10 had NW = 28 (93% of windows accepted for TWA analysis), and 2 PTI out of 10 had NW = 24 (80% of windows accepted for TWA analysis). ECG windows rejection was due to the presence of ectopic beats and not to high levels of HRV. TWA was found to be variable among our PTI (TWAA varied from 10 to 40 µV; Table 2). Correlation values between TWAA and clinical features at birth as well as additional ECG features are reported in Table 3. Correlation values between TWAA and BW, TWAA and GA/BW, and TWAA and HRV were found to be statistically significant ($\left|\rho \right|>0.70$, *p* < .05). The regression line between TWAA and GA/BW is depicted in Figure 3.

**Table 2:** T‐wave alternans and additional electrocardiographic features relative to a 1‐min recording of our ten healthy preterm infants

PTI	TWA features		Additional ECG features						
	NW (%)	TWAA (µV)	HR (bpm)	RR (ms)	HRV (ms)	QRSD (ms)	TA (µV)	QT (ms)	QTc (ms)
1	30 (100%)	16	163	369	8	85	258	285	469
2	30 (100%)	10	117	514	11	120	57	340	474
3	28 (93%)	18	114	525	43	95	168	360	497
4	30 (100%)	40	163	369	4	105	170	265	436
5	28 (93%)	26	171	350	20	95	504	295	499
6	30 (100%)	38	135	445	8	90	176	300	450
7	30 (100%)	39	182	329	8	100	328	265	462
8	30 (100%)	29	160	376	10	105	372	270	440
9	24 (80%)	32	138	436	7	90	132	325	492
10	24 (80%)	12	150	400	23	105	95	285	451
Tot	28 ± 2 (95 ± 8%)	26 ± 11	149 ± 23	411 ± 67	14 ± 12	99 ± 10	226 ± 139	299 ± 33	467 ± 23

Abbreviations: ECG, electrocardiogram; HR, median heart rate; HRV, heart‐rate variability; NW, number of windows; PTI, preterm infants; QRSD, median QRS‐complex duration; QT, median QT‐interval duration; QTc, median corrected QT‐interval duration; RR, median RR interval; TA, median T‐wave amplitude; TWAA, mean T‐wave alternans amplitude.

**Table 3:** Correlations of T‐wave alternans with clinical and additional electrocardiographic features of our healthy preterm infants

	TWAA	
	*ρ*	*p*
Clinical features		
GA	−0.46	.19
BW	**−0.72** *	**.02**
GA/BW	**0.76** *	**.02**
Additional ECG features		
HR	0.41	.24
RR	−0.41	.24
HRV	**−0.71** *	**.02**
QRSD	−0.17	.65
TA	0.42	.23
QT	−0.52	.12
QTc	−0.44	.20

Abbreviations: BW, birth weight; ECG, electrocardiogram; GA, gestational age; HR, median heart rate; HRV, heart‐rate variability; QRSD, median QRS‐complex duration; QT, median QT‐interval duration; QTc, median corrected QT‐interval duration; RR, median RR interval; TA, median T‐wave amplitude; TWAA, mean T‐wave alternans amplitude.

* *p* < .05.

The use of bold is to highlight the statistical significance.

## 4 DISCUSSION

The present work identified and quantified TWA in a population of ten nonpathological PTI. Such limited number is motivated by the deliberate selection of PTI without any comorbidity, who are statistically very difficult to obtain in clinical practice. In our PTI, TWA was evaluated in association with clinical features at birth and with additional ECG features that, in adults, are believed to be linked to TWA (Burattini et al., 2012; Hanna & Ahmed, 2013; Madias, 2007; Rashba et al., 2002; Zareba et al., 1994). The purpose is to gain some insights for better interpreting TWA in nonpathological PTI and, in particular, to open an issue about interpretation of TWA in nonpathological PTI as a heart‐developing marker rather than an arrhythmias‐risk index.

TWA was detected using the HRAMF method (Burattini et al., 2008), a widely tested method that can adapt to any heart rate and has proved to be robust to physiological levels of HRV and to presence of noise and interferences (Burattini et al., 2009; Burattini et al., 2011). HRAMF has been recursively applied to 64‐beat ECG windows in order to minimize rejections due to presence of HRV. Additionally, only 1 min of ECG was analyzed (although recordings were much longer) to provide a procedure easily applicable also when only short ECG recordings are available. Analysis of time variability of TWA in PTI will be matter for future studies. Additionally, only one ECG lead was available, and consequently, being TWA a lead‐dependent phenomenon (Burattini, Man, Burattini, & Swenne, 2012), the obtained TWA measures possibly represent an underestimation of the phenomenon.

Use of the HRAMF method also avoid false‐positive TWA measurements possibly due to the presence of QRS alternans, as well as other interferences or noise occurring at the same frequency. Indeed, the HRAMF method performs a strict filtering around the alternans frequency (AF, defined as half heart rate) and provides an output signal, termed the TWA signal, which is a pseudo‐sinusoid. Successively, the procedure performs a check on the TWA signal to locate all its zero‐derivative points (maxima and minima). If the maxima and the minima occur in correspondence of the T waves, TWA is actually detected; differently, TWA is set to zero, but other kinds of alternans, such as QRS alternans, may occur. Maxima and minima positions correspond to the location of the center of mass of all alternans affecting any ECG wave, thus including TWA and QRS alternans (Marcantoni et al., 2020). For example, if TWA is dominant or is the only type of alternans present, maxima and minima will occur in correspondence of the T wave. If QRS alternans is dominant or it is the only type of alternans present, maxima and minima will occur in correspondence of the QRS complex. Eventually, if TWA and QRS alternans are both present and comparable, maxima and minima will occur in an intermediate position between T wave and QRS complex.

For the specific final goal of this study, we only focused on TWA amplitude quantification. Thus, we did not window the QRS in order to detect QRS alternans, which could have been present but not dominant. Future studies will go deeper in this aspect and will attempt to discriminate various types of alternans, possibly preprocessing the ECG by setting to zero all waves but the one under analysis.

The first evidence of this retrospective study is that PTI show TWA, even without showing any other evident cardiac diseases. Levels of TWA affecting PTI are variable among subjects, similarly to what happens in adults (Burattini, Zareba, & Burattini, 2009, 2010) .

In our PTI population, TWA was found to decrease with BW (*ρ* = −0.72, *p* = .02) and to increase with GA/BW (*ρ* = 0.76, *p* = .02), thus indicating that TWA is higher in very small infants, especially when considering their weight in relation to their gestational age at birth. Additionally, TWA inversely correlated (*ρ* = −0.71, *p* = .02) with HRV, which is known to be a sign of health but also of growth and maturity (specifically, reduced HRV is a symptom of a late development of the autonomic cardiac control) (Kojima et al., 2018); indeed, HRV represents the heart ability to adjust to varying conditions. These findings suggest that observed levels of TWA in nonpathological PTI may still indicate a condition of cardiac risk possibly related to the low development status of the infant (low BW and low HRV) and, thus, an indirect marker of heart developing. This conclusion is supported by the results of a comparison between TWA levels in our PTI against term healthy fetuses. Comparing TWA levels among studies is always very difficult because TWA measurements depend on the method used to obtain them (Bini & Burattini, 2013). Nevertheless, in a previous study on healthy fetuses (Marcantoni et al., 2018, 2017) TWA was quantified at birth by using the same exact procedure used here. The age at birth of these fetuses varied between 38 and 41 weeks of gestation; thus, they were actually older than the PTI analyzed here (Table 1). TWA values observed in term healthy fetuses were around 10 µV (9 ± 2 µV when measured in direct transvaginal ECG acquisitions and 11 ± 5 µV in indirect abdominal ECG acquisitions), on average smaller than those observed in our PTI (Table 2). Thus, TWA seems to have the tendency to decrease with age at birth, finding that reinforces our hypothesis that observed levels of TWA in preterm newborns should probably be considered as an ECG manifestation of heart development rather than of severe heart pathology. Given the small size of subjects involved in the study, this conclusion should be considered preliminary. Further investigations dealing with wider populations of term and preterm infants should be performed in future studies. In adults, TWA associates to heart rate and QT length and represents an index of propensity to arrhythmic events (Burattini, Bini, & Burattini, 2012; Madias, 2007; Nearing & Verrier, 2002; Rosenbaum et al., 1996). Differently, in our nonpathological PTI, TWA does not correlate with HR neither with QT (Table 3), thus suggesting a possible different origin of the electrophysiologic phenomenon. Also, this other preliminary observation needs further studies to be confirmed. Indeed, a low correlation with HR may be due to the small population size and to the low difference of HR values among PTI (low HR standard deviation). Moreover, in this case, TWA is a spontaneous phenomenon, *that is,* not induced by increased HR reached through pacemaker or exercise (Burattini, Man, et al., 2012; Nearing & Verrier, 2002; Rosenbaum et al., 1996).

In conclusion, our study on ten nonpathological PTI provided some insights into the TWA phenomenon in preterm infants and opened an issue on its interpretation that requires further investigations to be solved.

## CONFLICT OF INTEREST

All authors have no financial and personal relationships with other people or organizations that could inappropriately bias the work.

## AUTHOR CONTRIBUTIONS

Ilaria Marcantoni designed the study, developed the conceptual method, interpreted the results and drafted the manuscript. Agnese Sbrollini helped in the statistical analysis and in the interpretation of the results. Gloria Agostinelli computed the QT features. Francesca Chiara Surace and Massimo Colaneri gave their contribution in the clinical evaluation and interpretation of results. Micaela Morettini helped in the statistical analysis and in the drafting of the paper. Marco Pozzi supervised the clinical aspects of the work and critically revised the manuscript. Laura Burattini supervised the technical aspects of the work (feature computing, algorithm implementation, statistical analysis) and critically revised the manuscript. All the authors gave final approval of the manuscript.

## ETHICAL APPROVAL

Data used in the present study are available from Physionet. All data from Physionet have been fully de‐identified and may be used without further independent ethics committee approval.
